# Early introduction of 3D modeling modules promotes the development of simulation skills in downstream biomedical engineering curricula

**DOI:** 10.1186/s13036-023-00339-7

**Published:** 2023-03-30

**Authors:** Mary S. Jia, Raj R. Rao, Mostafa Elsaadany

**Affiliations:** Department of Biomedical Engineering, University of Arkansas, Fayetteville, USA

**Keywords:** Simulations, 3D Design, SolidWorks, Engineering Pedagogy, Biomechanics

## Abstract

**Background:**

Recent advancements in additive manufacturing have made 3D design a desirable skill in combating the historically slow development of biomedical products. Due to the broad applicability of additive manufacturing to biomedical engineering, 3D design and 3D printing are attractive educational tools for biomedical engineering students. However, due to the multidisciplinary nature of biomedical engineering, finding a suitable spot in the curriculum to teach students basic and application-based skills for 3D manufacturing is difficult. Furthermore, prior training in fundamental 3D design skills may be needed to support the use of application-based supplementary content.

**Results:**

We designed a SolidWorks Simulations toolkit to complement a sophomore (2nd-year)-level Biomechanics course and distributed this assignment to students with and without prior training in 3D design delivered in an introductory biomedical engineering course. Using short videos, example-based problem solving, and step-by-step tutorials, students completed this as an extra-credit assignment and completed a survey gauging student opinion on SolidWorks and 3D design, confidence in each target skill, and the effectiveness of assignment delivery. The compilation of survey responses suggests that the assignment effectively increased positive responses in student opinion on interest in and likeliness to use SolidWorks in both groups. However, confidence in the target assignment skills was higher in the trained group and fewer problems occurred in operating SolidWorks for trained students. Further, analyzing the distribution of student grades with respect to survey responses suggests that responses had no relationship with initial class grade.

**Conclusion:**

These data collectively indicate that prior training provided to the students had a positive impact on the effectiveness of the assignment although increases in student opinion on the utility of 3D design were observed in both trained and untrained students. Our work has generated and identified a useful educational supplement to enrich existing biomedical engineering course materials with practical skills.

**Supplementary Information:**

The online version contains supplementary material available at 10.1186/s13036-023-00339-7.

## Introduction

Additive manufacturing, or 3D printing, enables the cost-effective, precise, and rapid synthesis of complex 3D structures and is increasingly supplanting many conventional 3D manufacturing methods. The biomedical industry has also experienced the impact of this revolution in device and material development. In recent years, biofabrication with 3D printing has been mobilized to replicate complex physiological environments, generate implantable devices or biomaterials, and manufacture drug delivery systems. The construction of microfluidic organ-on-a-chip devices has successfully demonstrated that bioprinted constructs can produce biomimetic in vitro 3D microenvironments with translational drug discovery applications [1–3]. Furthermore, the fabrication of drug delivery devices such as tablets with controlled release pharmacokinetics is additionally experiencing expedited development facilitated by 3D printing [4, 5]. Rapid prototyping through CAD software and 3D printing technologies is also being used for the development of implantable biomaterials and orthopedic implants [6]. The usage of bioprinting and 3D printing in the biomedical field is expanding to combat the slow development period for biomedical products.

With the advent of 3D printing for biomedical applications such as in biomaterial development and in complex physiological models through organ-on-a-chip, the necessity of providing biomedical engineering (BMEG) students with training in 3D design techniques is becoming an increasingly relevant concern. BMEG, as the fastest growing engineering subdiscipline, presents a difficulty in developing pedagogical methods consolidating both the biomedical sciences and engineering principles in addition to a sufficient education in supplementary necessities such as ethics [7]. Integrating applications that supplement students exiting their undergraduate program with desirable skills provides further impetus to alloy offered theoretical content with an active practice. Current additive manufacturing trends in biomedical industries support providing training for BMEG design professionals in 3D design software but do not require such training for entry [8]. Biomedical engineers are expected to possess or be able to quickly obtain sufficient 3D design knowledge for biomechanical applications and medical device development, but the implementation of 3D design in BMEG courses is less prevalent compared to alternative engineering subdisciplines partly owing to the broad scope of the field. Most biomedical engineering educators presume that students are autodidactic due to the ease with which software such as AutoCAD and SolidWorks may be operated and would not benefit from 3D design instruction. However, there is little evidence supporting this assumption, and the perceived competency of BMEG graduates that are self-taught in 3D design could potentially be a product of a significant amount of time spent practicing or learning through online courses. Due to the increasing popularity of 3D design in the BMEG discipline, integrating 3D design through computer-aided design (CAD) -based services in engineering courses is expected to support the preparation of engineering students for their future careers [9, 10]. It is, then, necessary to determine whether there is a tangible benefit from including 3D design training in the BMEG curriculum.

Criticisms of the restrictiveness of a curriculum contingent on 3D design elements have been raised, warning educators against restricting imaginative and innovative thinking [11]. Designing and running simulations on models in 3D design software such as SolidWorks follows a linear and structured design process that produces an easily visible result. Distinctions between the cognitive processes leading to a routine vs. an innovative product have indicated that generating students trained in a linear design-to-product workflow reduces the capacity for adaptive problem-solving [12]. Furthermore, over-reliance on CAD programs with implicit limitations can cause the engineer to focus too much on optimizing functionality within the confines of the software, which may not translate to actual physical behavior [11]. Combining SolidWorks design and simulation with the creative process as a supplement rather than a pillar for general design is positively associated with increasing creative motivation and reducing bounded ideation [11]. Here, we suggest that integrating 3D design-related modules into existing course material can augment the development of desirable 3D design skills while avoiding truncating foundational course material and inhibiting creative thought. SolidWorks Simulations offers simulation software for fluid flow, thermal, vibrational, and static simulations, which provides a broad range of skills for research or product design. However, the ability of students to navigate and generate results in the SolidWorks Simulations software is contingent on the autodidactic capacity of the student to learn basic 3D design or previous experience in operating 3D design software. Providing two groups of students with a module integrating 3D design within a downstream BMEG course that has and has not been provided with basic 3D design training and in-class practice can confirm whether initial training would benefit students in later applying 3D design skills.

In this study, we investigated the impact of preexisting introductory 3D design training on students' pre-conceived notions of CAD and the ability of students to generate 3D simulations in SolidWorks. This study was performed on a cohort of students that experienced a traditional introductory BMEG course in their freshman (1st) year and a second cohort that was given a SolidWorks introductory module in the Introduction to BMEG course. The Biomechanics sopohomore (2nd-year)-level course was the target to integrate 3D design simulation elements, which is primarily due to the problem sets for the course consisting of basic statics parameters. A simulation toolkit composed of 14 static simulations of homework problems and lecture examples was distributed with short videos guiding students through example simulation problems. Through the assignment, students would be able to familiarize themselves with SolidWorks design elements and simulations in addition to applying 3D visual learning to familiar biomechanics problems. This allows students to compare the simulation results to the analytical -by hand- solution of in-class problems due to the at times inaccurate assumptions of finite element analyses. Feedback from before and after the assignment was solicited through a pre- and post-assignment survey to gauge student confidence in operating the software, assess student interest and knowledge in 3D design, evaluate the effectiveness of the assignment delivery, and determine whether students experienced technical errors that occurred in accessing the assignment. This study aimed to accomplish an assessment of whether incorporating a SolidWorks Simulations component in the Biomechanics course could facilitate students’ ability to produce and analyze 3D models and whether previous basic 3D design training in the SolidWorks software would improve the effectiveness of 3D design integration at an ABET (Accreditation Board for Engineering and Technology)-accredited university.

## Methods

### Course design and 3D design element integration target

**Fig. 1:**
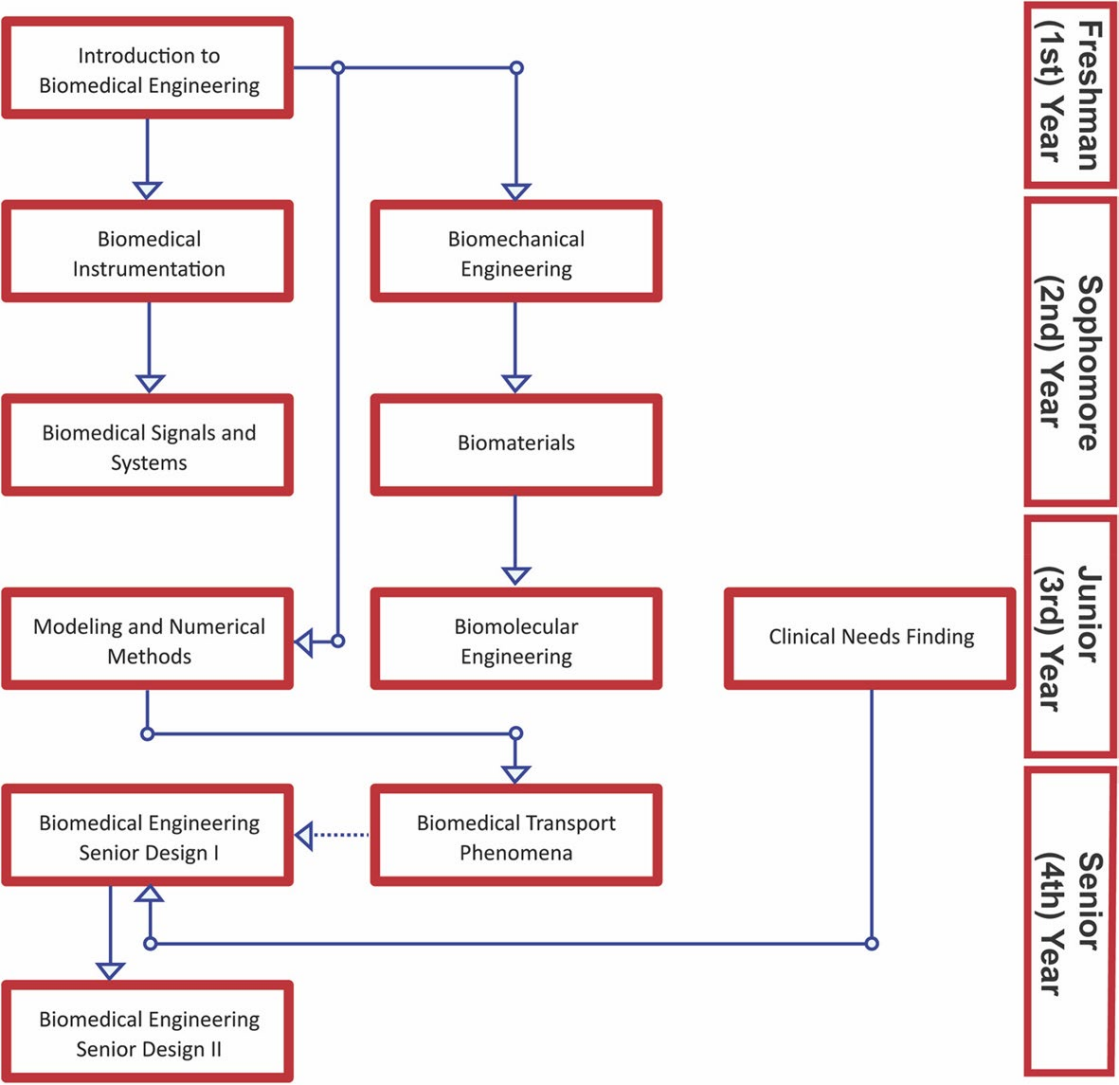
Basic coursework outline for BMEG students reduced to the BMEG-specific courses alon e

The target course for applying a simulation component to the original coursework was the sopohomore (2nd-year) second-semester Biomechanical Engineering course (Fig. 1). The justification for selection was due to the simplicity through which many of the basic statics biomechanics problems can be modeled in the SolidWorks software. Furthermore, students would be able to visualize the result of the loads we are simulating on simple beam systems, which we hypothesize will improve their conceptual understanding of the equations used in the course.

The first cohort of students given the option to complete an extra credit assignment containing the Simulation Toolkit had worked through the original curriculum lacking SolidWorks training. However, in the second cohort of students that were enrolled in the Biomechanics course the subsequent year, an introductory basic SolidWorks component was briefly included in the Introduction to Biomedical Engineering course, which surveys the field to provide students with a broad perspective of the discipline.

### Introductory SolidWorks module

**Fig. 2:**
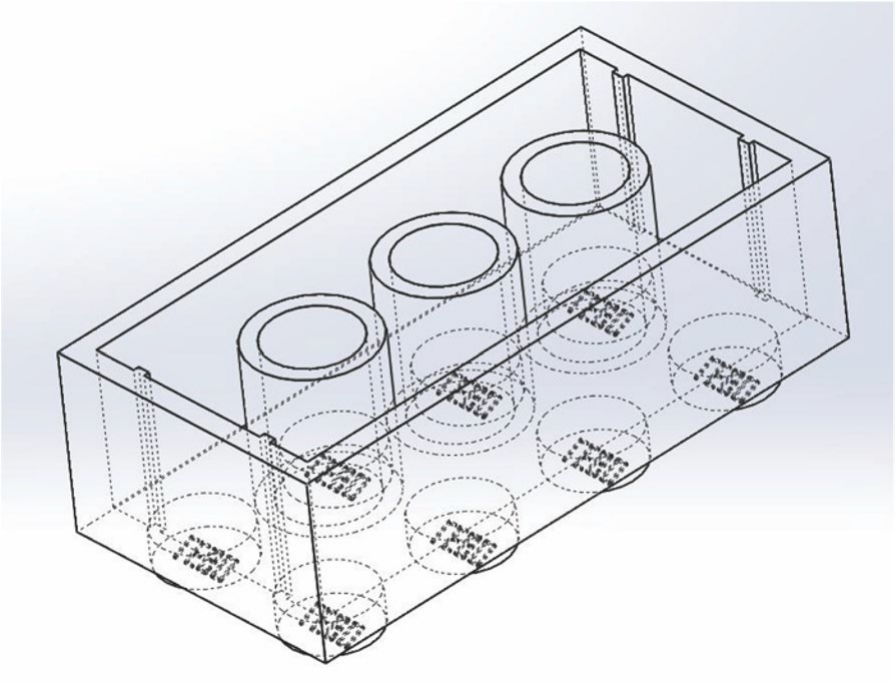
Schematic of the Legos block students were asked to generate in the Introduction to Biomedical Engineering course as an introductory component to SolidWorks modeling

A second cohort of students was provided with training in SolidWorks basic modeling incorporated in their Introduction to Biomedical Engineering course, which was taken a semester prior to the Biomechanics course. This assignment was provided as an optional grade to replace their lowest homework grade at the end of the semester. The basic modeling tutorial required students to generate a functional Legos block with a fun fact about themselves written on the bottom of the model and a drawing illustrating multiple angles of the model (Fig. 2).

### Content delivery method

The resulting Simulation Toolkit contains 14 similar statics simulations and 5 videos including a tutorial for accessing and navigating SolidWorks and three step-by-step example problems were included in a Box file and distributed to students. Box is a cloud-based file storage application that all students have access to and is an effective method of storing and sharing large files such as SolidWorks parts and simulation files. Notably, the second cohort of students that had taken the introductory SolidWorks module were also provided with written step-by-step instructions in addition to the demos based on the previous cohort reporting difficulties in following along with the video instructions, which does confound conclusions made in the post-assignment survey. Introductory demo videos instructed students on how to access SolidWorks on their computers. Students could access SolidWorks by (1) downloading the student license provided by the University on a windows-running computer or (2) through the Citrix Workspace cloud computing app. If their computer was insufficient for running the software or if they were encountering persisting errors, the students were invited to complete the extra credit assignment in an on-campus lab with computers capable of running SolidWorks. Students were also encouraged to explore and attempt running any remaining simulations provided in the toolkit.

**Fig. 3:**
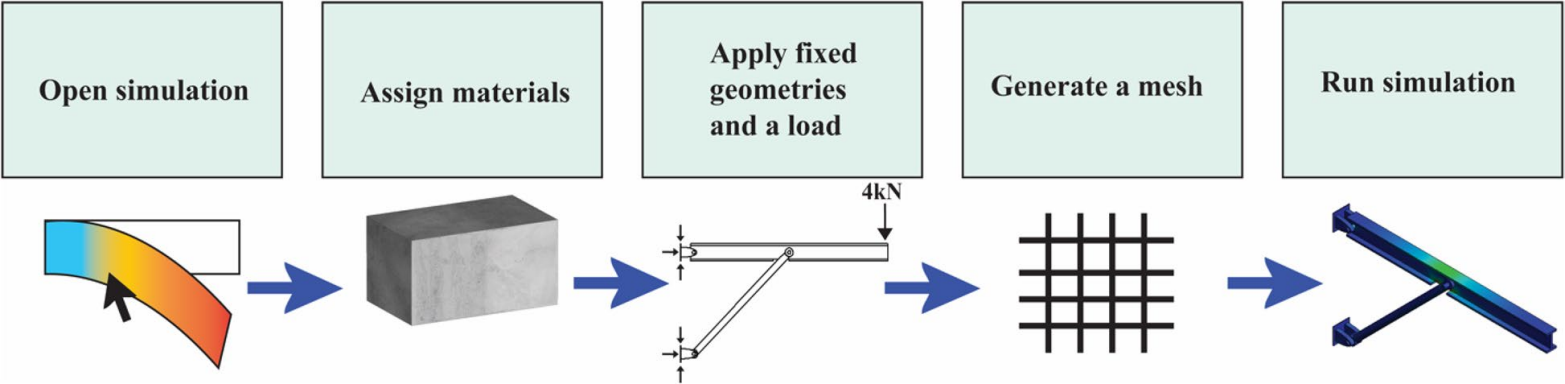
The general statics workflow provided in the video demos and written instructions for students to parameterize and run static simulations in SolidWorks Simulations with provided 3D models

The instructions used to train students to run and analyze the example simulations was a linear process requiring critical thought at specific steps (Fig. 3). The demos were designed such that students could watch the video while parameterizing their own static simulations. Depending on the model, the students would need to adjust the FEM solver and meshing to increase the speed at which the simulation is solved by the SolidWorks software. Additional critical thinking was required for students to determine how they to accurately apply the load listed in the problem statements using different annotation methods. If students were unable to replicate the simulation, a completed static study was also provided with the correct simulation solution in the same files. Students uploaded images of their simulation results and comparisons to analytical solutions, and a completion grade was given to participating students as extra credit. Optional instruction was provided through teaching assistant office hours and at the student’s request.

### Designing SolidWorks simulations

We derived the 3D SolidWorks models here from homework and lecture problems in the Biomechanics course, including moment, stress, axial loading, bending, shear, beam deflection, and static equilibrium problems. The SolidWorks Simulations software statics parameters generated by the students determine the displacement or stress values in each element of a discretely approximated object connected by a pre-defined coarseness of nodes through the finite element method (FEM). For students to define the exact bounds of an individual element, a mesh defining the polygonal representation of the object, including the number of nodes, and physical restrictions was applied by the students to the model before the simulation study was run. After running the simulation study the students independently designed and restricted, the students can determine the value of variables such as bending stress as requested by the problem statement by probing for values at specific locations. As most problems in the introductory Biomechanics course are 2D, additional approximations were made to convert models into a 3D problem in the models by the instructor, which further increases discrepancies between the 2D static solution and simulation result. These approximations were incorporated in the model supplied to students to allow them to focus on generating the simulation rather than developing a suitable model. Supports that are assumed rigid in the static solutions are assumed to be composed of the same material and simply supported objects are assumed to be geometrically fixed. Students were asked to enforce these assumptions in generating the simulation parameters and restricting the model to provide them with perspective on the degree of assumptions needed to obtain their simulation data and corresponding potential sources of simulation errors.

**Fig. 4:**
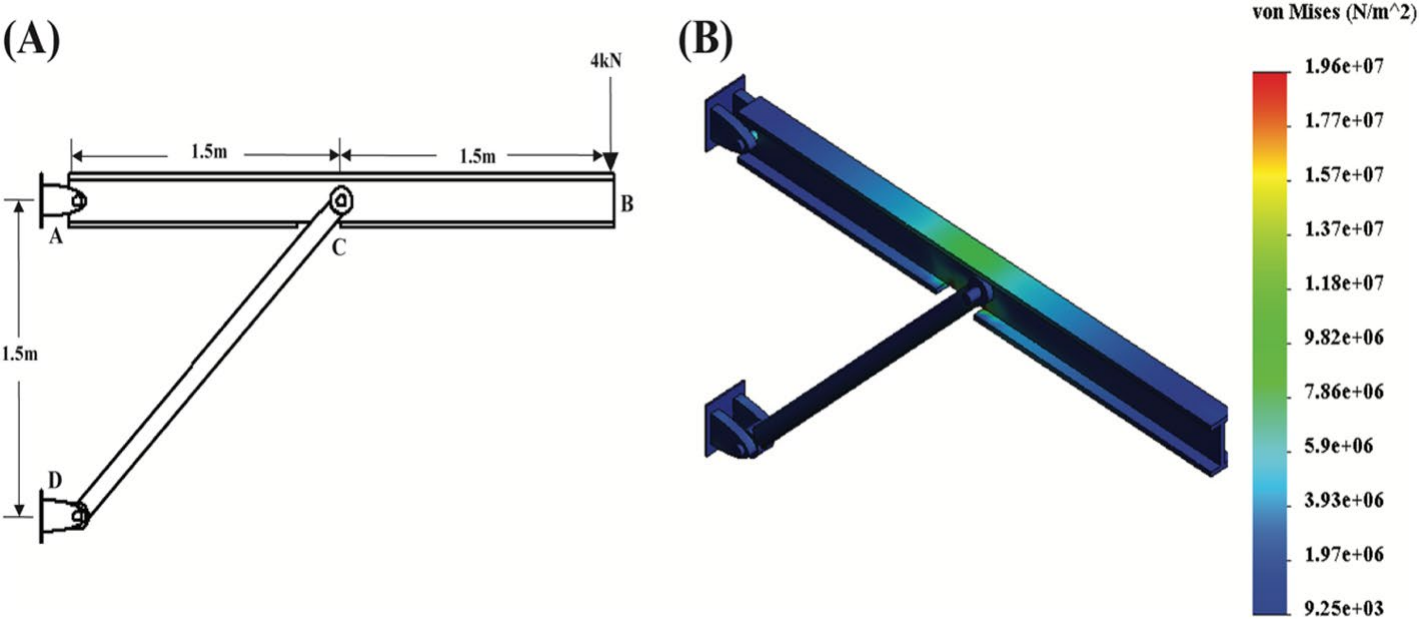
**A** A36 steel beam system under a 4kN load as described in a lecture example. **B** Simulation output where students can probe for reaction forces located at supports A and D in SolidWorks and compare the simulation results to analytical solutions

Students were provided with three demo problems asking the student to derive support reactions, confirm that a uniform distributed load does not exceed a given maximum bending stress, and determine displacement due to a point force, respectively. Students would determine support reaction FEM estimations by defining the face at which a result force should be calculated in SolidWorks Simulations. As an example of the types of problems designed for the simulation toolkit, the first problem (Fig. 4a) is regarding a beam supported by pins that is experiencing a 4kN load and originates from a lecture example. Students are asked to determine support reactions using static equilibrium analytically and, using SolidWorks simulations, to compare that result to the simulation solution derived by probing for force values at each support. The displacement of an object and the von Mises stress were similarly determined in the SolidWorks Simulations software using the probe results function to output a graph of the stress along the defined parameterized geometry of an object (Fig. 4b). The von Mises stress accounts for the maximum distortion energy theory, which states that once the distortion energy per unit volume experiencing non-uniaxial stress or non-simple tension load becomes equal to the yield stress in a uniaxial tensile test at any point in the object, failure by yielding may occur [13]. The von Mises criterion for yield more accurately models complex loads applied to ductile objects, which is often the case in problems used in the Biomechanics course. The resulting displacement calculations are additionally impacted by the usage of the von Mises criterion.

**Fig. 5:**
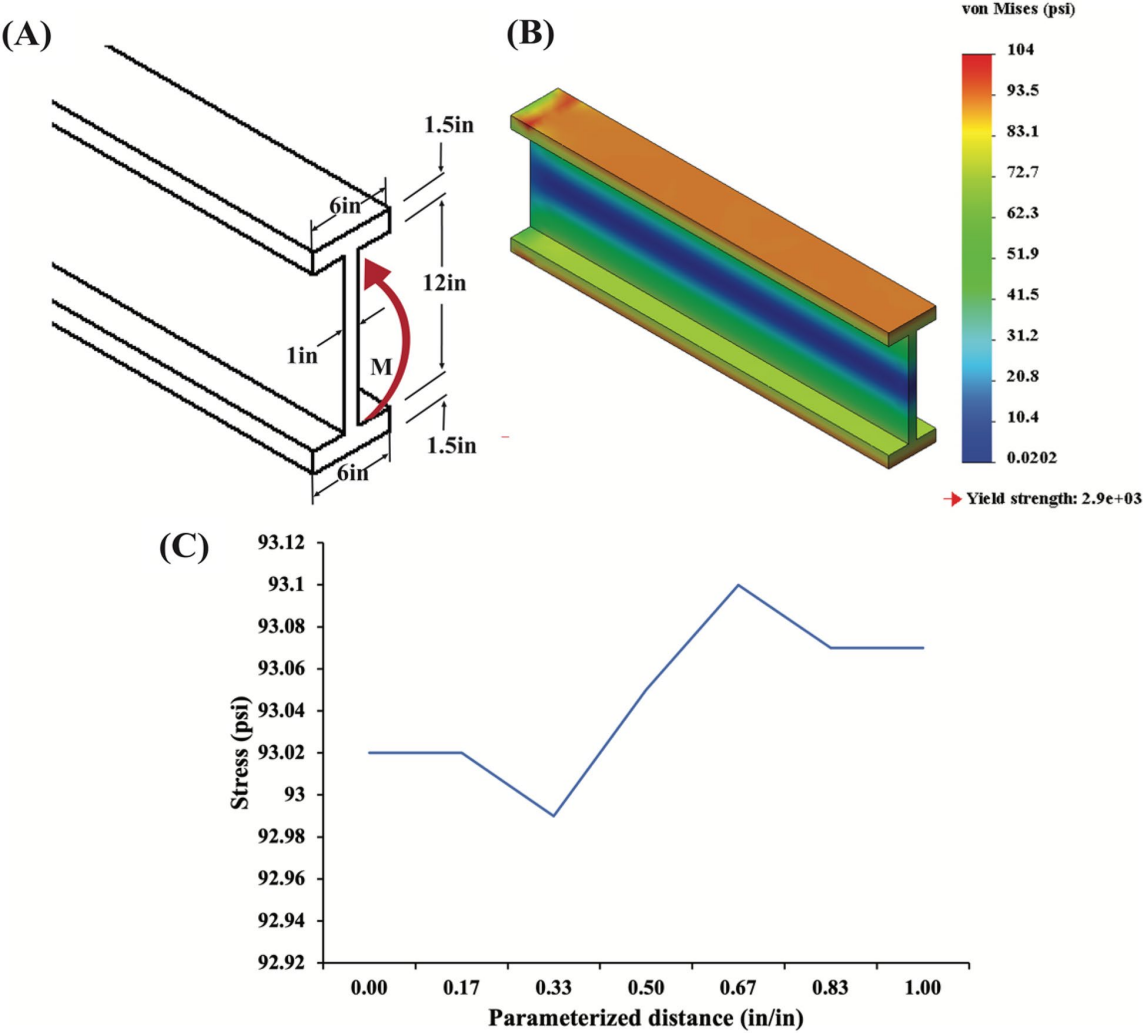
**a** A36 steel I-beam under a moment force in the cross-sectional area as described in a homework problem. **b** Simulation output where students can probe along the top edge of the cross-section and ascertain maximum stress to compare with analytical solutions. **c** The result derived by probing for stress values along the top edge of the I-beam cross-section where a moment is applied

The second example (Fig. 5a) is from a homework problem that asks students to determine the maximum bending stress in the cross-sectional area of an I-beam experiencing a 1 kip*ft moment (Fig. 5a and b). Students were asked to compare the results from FEM simulations to the analytical solution and to compute an error percent between both solution methods, which was determined to be 0.11% (Fig. 5c). Based on this result, students were allowed to draw their own conclusions regarding the discrepancy between calculation methods. This assignment structure permits a progressive understanding of the method of designing a simulation model while providing a visual and conceptual supplement to existing course concepts.

### Data collection from students

The surveys and experiments conducted in this study were performed under formal approval from the University of Arkansas Institutional Review Board (IRB #2,012,306,631). A survey was distributed to students participating in the extra credit assignment through the Qualtrics software (Qualtrics Core XM). The questions included in the survey were sorted into three broad categories (Additional file 1): (1) previous experience with 3D design and CAD-based software, (2) an assessment of the effectiveness of instruction and the ease with which students could complete the assignment, and (3) interest and likeliness to use SolidWorks as a 3D design tool in a survey conducted before and after the assignment. These data were used to determine whether the layout and instructions used for the assignment were sufficient for students to be able to generate their own simulation studies. To account for the possibility that the written instructions only being provided to the trained cohort may have some influence in their responses, student perceptions were assessed post-assignment delivery in both cohorts. Student course grade distributions were also analyzed with respect to responses in the survey to determine whether initial grade influenced their response to confidence in skill or effectiveness of assignment.

### Statistical analyses of selected responses

Statistical analyses were performed using nonparametric tests due to the data being on a Likert-type scale that assigns a numerical value to ordinal data and were represented similarly [14]. A Kruskal–Wallis test was used to analyze statistically different responses based on deviations from the median rather than the parametric mean for multiple groups. After the Kruskal–Wallis demonstrated significance, a Dunn post-hoc was used to compare between each group. Additional tests assessing whether the data were normally distributed were performed (e.g., Anderson–Darling, Shapiro–Wilk, Kolmogorov–Smirnov, and D’Agostino & Pearson tests) to confirm the necessity to deviate from parametric statistics. For two groups, a Wilcoxon ranked sum test was used to assess significance. Student responses and course grades were also correlated to determine whether course standing impacted responses.

## Results

**Fig. 6:**
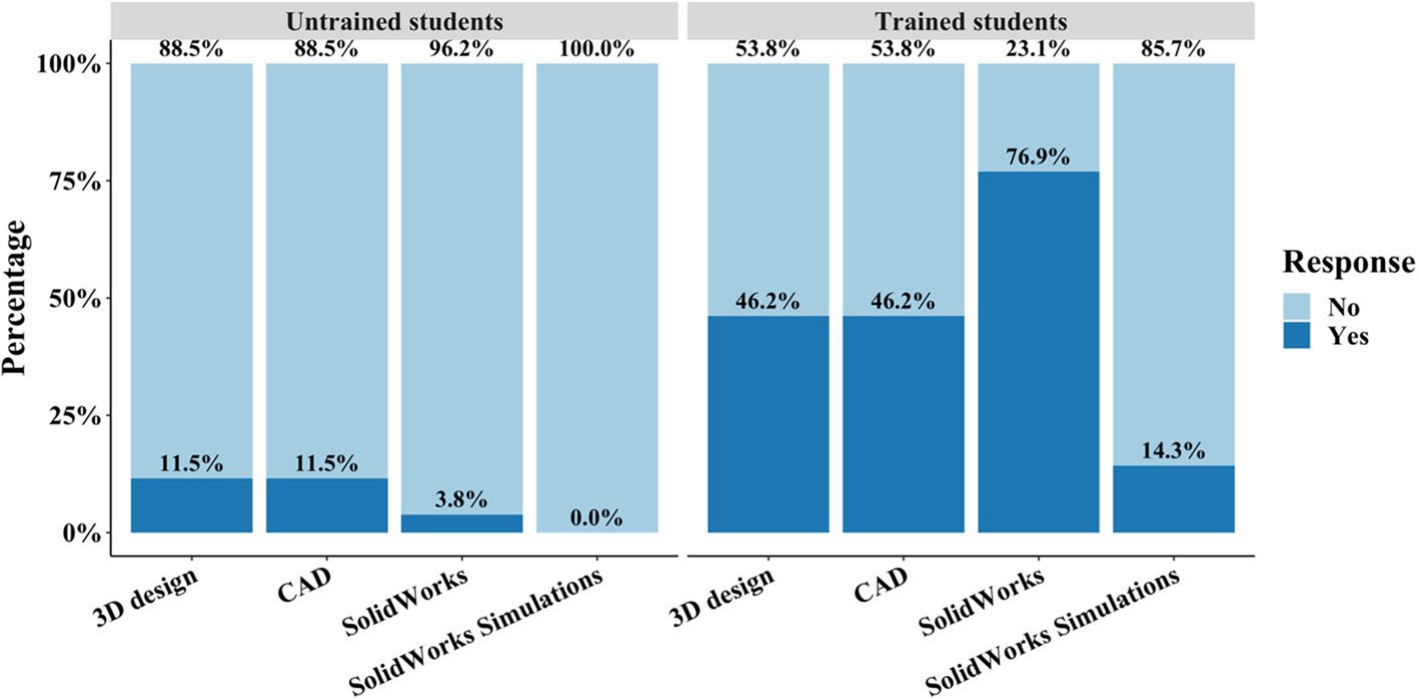
Distribution of student experiences in 3D design, CAD programs, SolidWorks, and SolidWorks simulations. In the untrained cohort, > 88% of students in the untrained cohort responded with no experience in 3D design. In the trained student a higher proportion of students indicated some training in 3D design skills

From the initial cohort of students without training, 26 students out of 51 total students participated in the extra-credit assignment while the second cohort with introductory SolidWorks knowledge only had 21 participants out of 55 students. For each student cohort, participating students had a 100% completion rate. Student responses gauging the previous knowledge and experience possessed in CAD-related software and simulations were assessed to determine a baseline for each cohort. In the untrained cohort, 88.5% of students had zero prior experience with any 3D design software while 11.5% had some experience in 3D design and CAD programs (Fig. 6). An even lower 3.8% of students reported experience in using SolidWorks and no students in the first cohort of untrained students had any experience with SolidWorks Simulations. Due to the SolidWorks module provided in the introductory course, all students in the second group had some experience in 3D design software with 76.9% of students reporting experience with SolidWorks in particular (Fig. 6). Furthermore, 14.3% of students reported experience in SolidWorks simulations from independent exploration outside of coursework. For each skill, differences for untrained students were statistically significant for 3D design, CAD, and SolidWorks (*p* < 0.05) while trained students had no statistical significance except for SolidWorks Simulations.

**Fig. 7:**
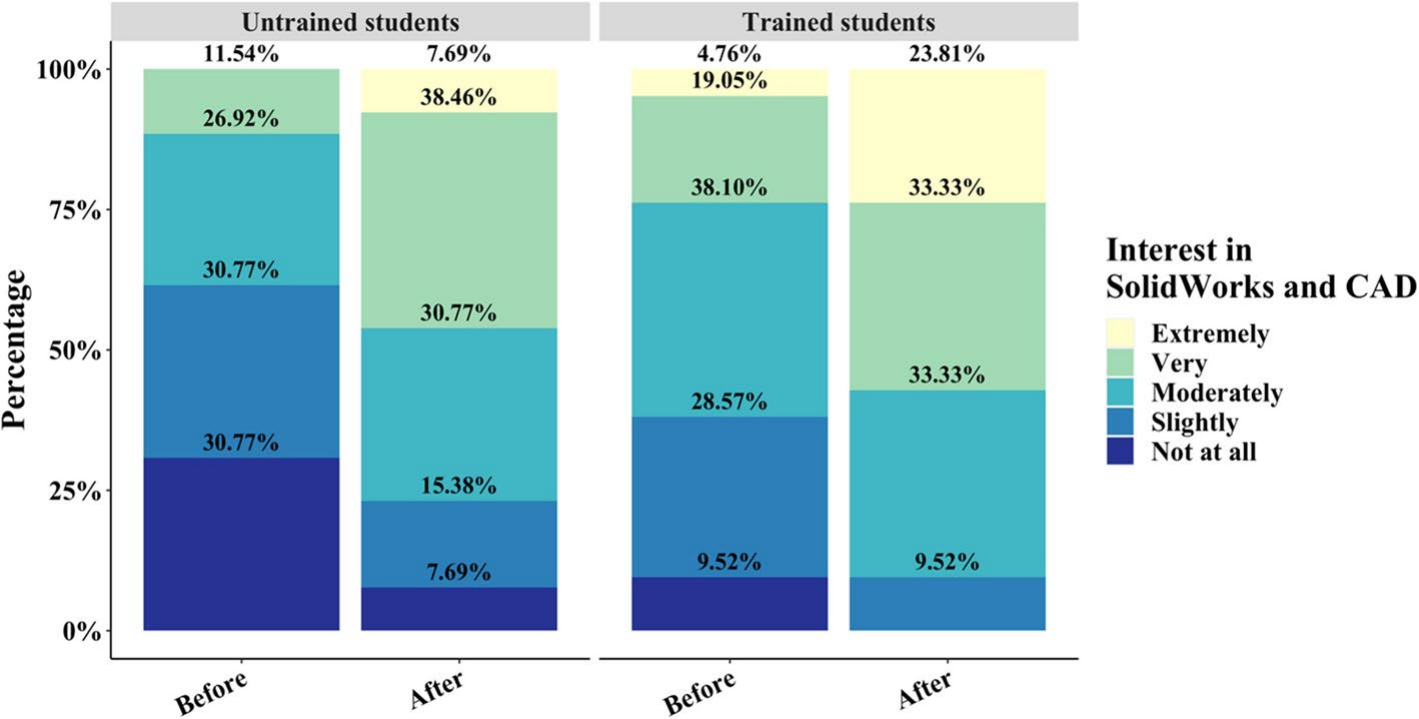
Likert plot of student interest in SolidWorks and CAD before and after completion of the assignment for untrained and trained cohorts

Likert-like data based on responses that allowed students to rate their likeliness, interest, confidence, or difficulty in completing an assignment are represented by assigning each response a number increasing in positivity from 1 to 5. Overall, 51% of untrained students in the Biomechanics course participated in the simulation assignment while 38% of trained students participated. A larger proportion of untrained students participated in the extra credit assignment, and further in-depth analysis of the degree of interest participating students expressed was further investigated using survey data. Student interest prior to the extra credit assignment in the untrained group majorly fell within the range of no interest to a slight interest in CAD-based software (Fig. 7). However, untrained negative responses, which is slight or no interest, collected after the survey decreased from 61.54% to 23.07%. Responses indicating that the student in the untrained group is very or extremely interested, which is considered a positive response, in CAD software increased from 11.54% to 46.15% indicating that untrained students that initially expected the assignment to be of no interest to their careers had responded with increased positivity upon completion of the assignment (*p* < 0.001). The trained cohort of students experienced a similar trend and distribution of students expressing an interest in SolidWorks and CAD. Students in the trained group decreased in negative responses from 38.09% to 9.52% after completing the assignment (Fig. 7). Positive responses in the trained group increased from 23.81% to 57.14%. Between the trained and untrained cohorts, there was no statistically significant difference in responses between these groups before or after completing the assignment based on Wilcoxon-ranked sum tests (*p* > 0.05).

**Fig. 8:**
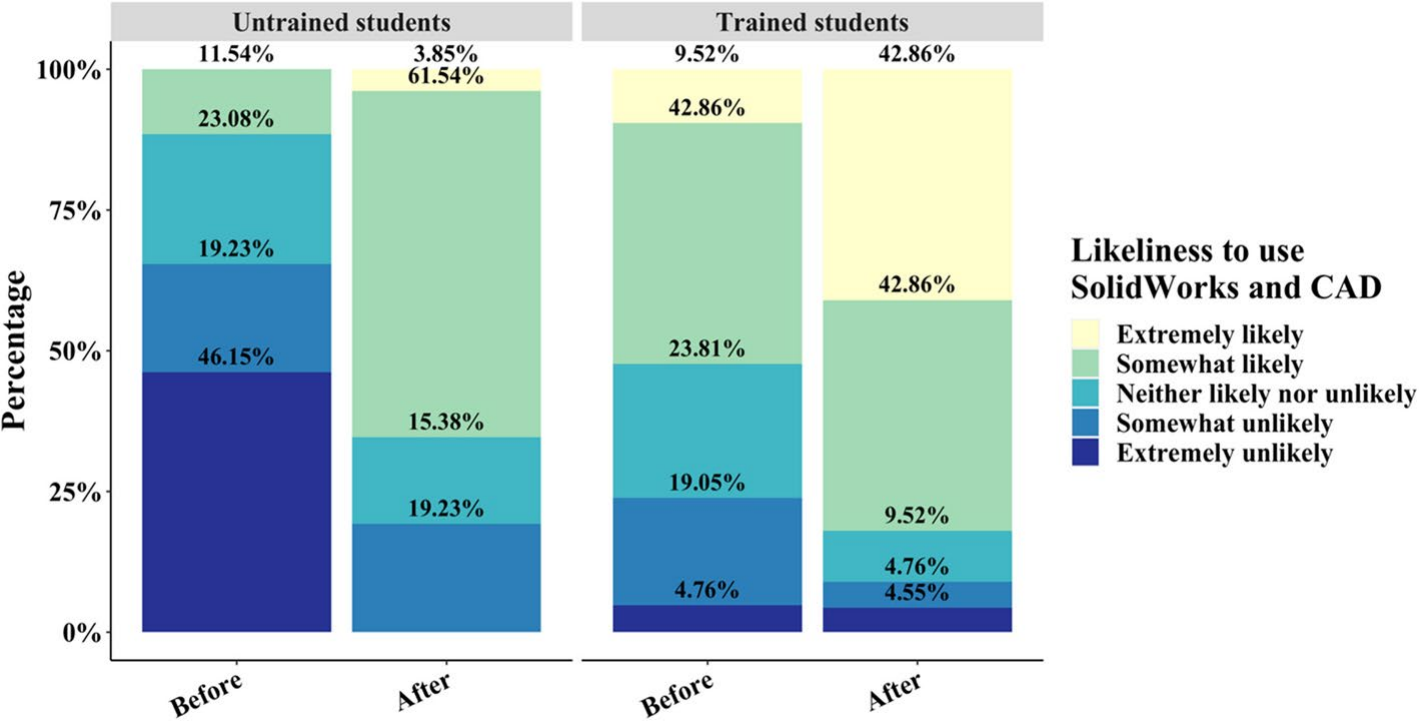
Likert plot of student likeliness to use SolidWorks and CAD in the future before and after completion of the assignment for the untrained and trained cohort. The likeliness to use SolidWorks and CAD increased in both groups with a significantly more severe increase both before and after assignment delivery in the trained cohort

The question gauging the likeliness of students to use SolidWorks in their future career followed a similar pattern in the untrained group with responses indicating extremely or somewhat unlikely decreased from 65.38% to 19.23% while responses indicating extremely or somewhat likely increased from 11.54% to 65.39% (*p* < 0.001) (Fig. 8). Interestingly, in the trained group, most students responded positively both before and after completing the assignment with 52.38% and 85.72% of students expressing extreme or somewhat likeliness to use SolidWorks and CAD, respectively (Fig. 8). The previously larger neutral group of 23.81% of students decreased to 9.52% and the students responding negatively decreased from 23.81% to 9.31%. Between the trained and untrained cohorts, trained students exhibited a statistically significant increase in likeliness to use SolidWorks and CAD both before (*p* < 0.001) and after (*p* < 0.01) assignment delivery.

**Fig. 9:**
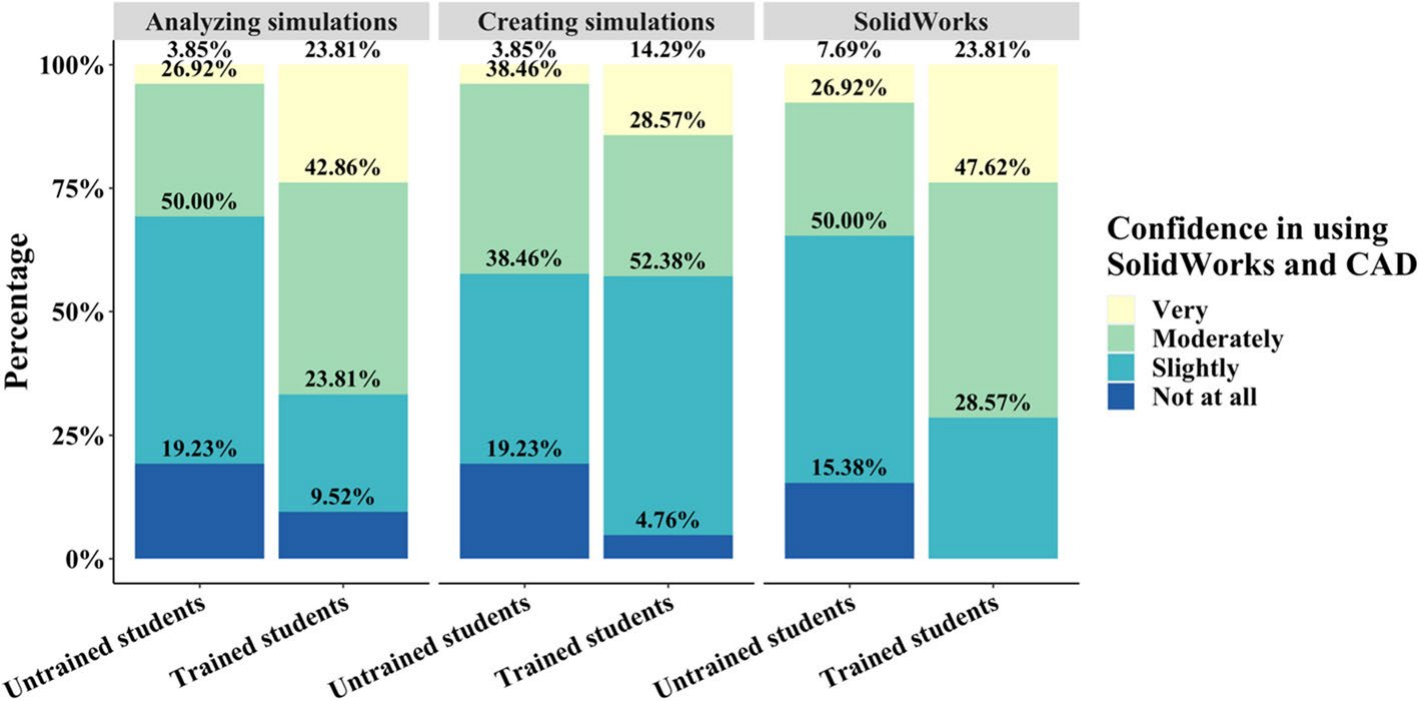
Likert plot of student responses assessing confidence in using SolidWorks and SolidWorks Simulation software in the post-assignment survey. Confidence in SolidWorks and analyzing simulations increased significantly in the trained group while creating simulations did not significantly differ

To diagnose the confidence in 3D design and simulation skills students had obtained upon completion of the simulation toolkit assignment, we also asked students to rank their confidence in each skill using a Likert scale. Student confidence in operating SolidWorks, analyzing simulations, and creating simulations between the trained and untrained groups demonstrated a stronger consensus in response compared to other questions. The student confidence in using SolidWorks, analyzing simulations, and creating simulations for the untrained cohort before was heavily biased toward 65.38%, 69.23%, and 57.69%, respectively, of students responding with no or slight confidence (Fig. 9). The trained students, however, responded with 28.57%, 33.33%, and 57.14% slight or no confidence in SolidWorks, analyzing simulations, and creating simulations, respectively (Fig. 9). A larger percentage of students in the trained cohort had responded neutrally to their strength of confidence in using SolidWorks and SolidWorks simulations compared to the untrained group of students. (Fig. 9). Based on a series of Wilcoxon tests between the trained and untrained cohorts, confidence significantly increased in SolidWorks (*p* < 0.01) and analyzing simulations (*p* < 0.05) in the trained group. Untrained and trained students exhibited similar confidence in creating simulations, indicating a deficiency in the assignment design for developing this particular skill. Internally, the untrained students were statistically significantly inclined to the slight or no confidence in all skillsets (*p* < 0.05), while the trained students exhibited no statistical significance in a Kruskal–Wallis test. These data indicate that confidence levels were more evenly distributed, but overall more positive, in the trained cohort.

**Fig. 10:**
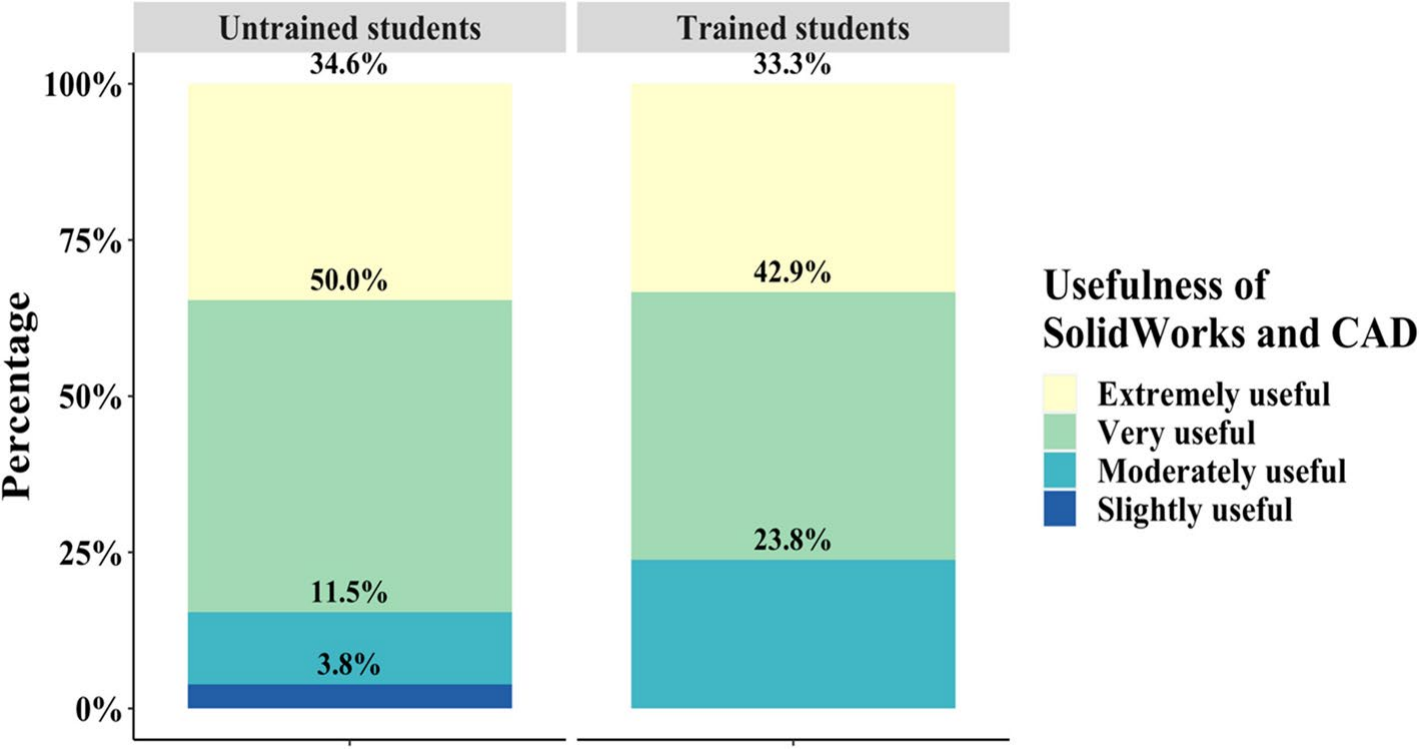
Student responses regarding their perception of the usefulness of SolidWorks Simulations after completion of the assignment revealed highly similar responses between trained and untrained students

Students assessed the usefulness of SolidWorks Simulations, and their ability to complete the assignment, and provided additional feedback after the assignment through the post-survey. Consistent with the increasing positive opinion of SolidWorks and CAD in previous questions, 84.6% of untrained students indicated that SolidWorks Simulations is a very or extremely useful tool (Fig. 10). The trained group responded with a slightly lower 76.2% of students responding positively and a neutral group consisting of 23.8% of students (Fig. 10). There were no negative responses to the usefulness of SolidWorks in the trained group while the untrained group had 15.3% of students expressing that SolidWorks is slightly or not useful. Based on a Wilcoxon test, there is no statistically significant difference between the two groups (*p* > 0.05). Internally, the trained students had no statistical significance between each group while the untrained students were significantly responded positively.

**Fig. 11:**
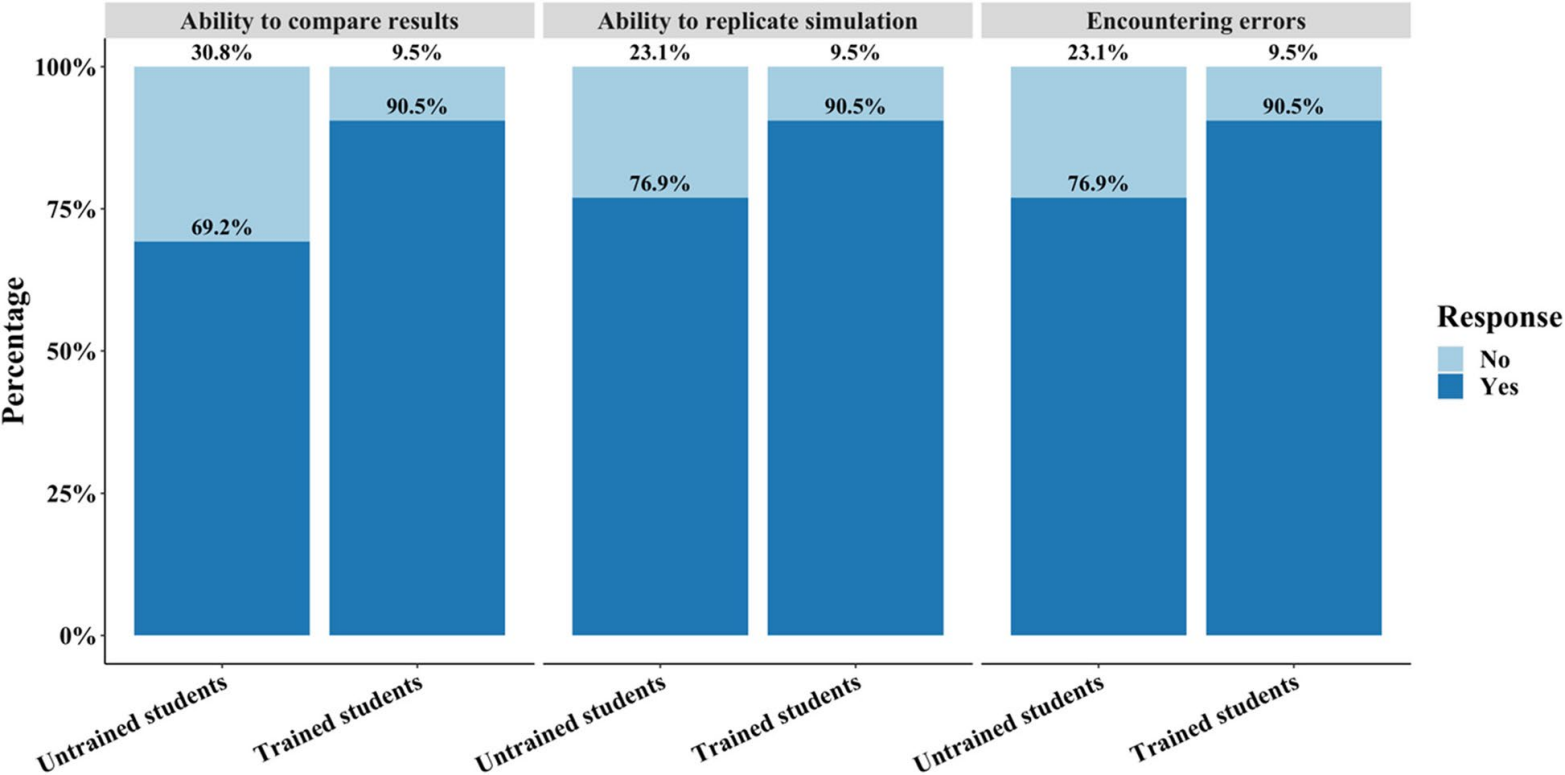
Likert plot of student responses in the post-assignment survey regarding the ability to compare simulation to analytical results, ability to replicate the simulation, and whether students encountered errors. The ability and errors encountered increased in the trained group in all areas but were not statistically significant

Most students participating in the study also encountered errors but were ultimately able to compare the results of the simulation with their theoretical solutions for the problem, which reduced in the trained group with a consistent 90.5% of students successfully completing the assignment with few errors (Fig. 11). However, responses regarding the ability to compare results, replicate simulations, and whether errors were encountered had no statistically significant differences using a Kruskal–Wallis between trained and untrained for each question (*p* > 0.05).

**Fig. 12:**
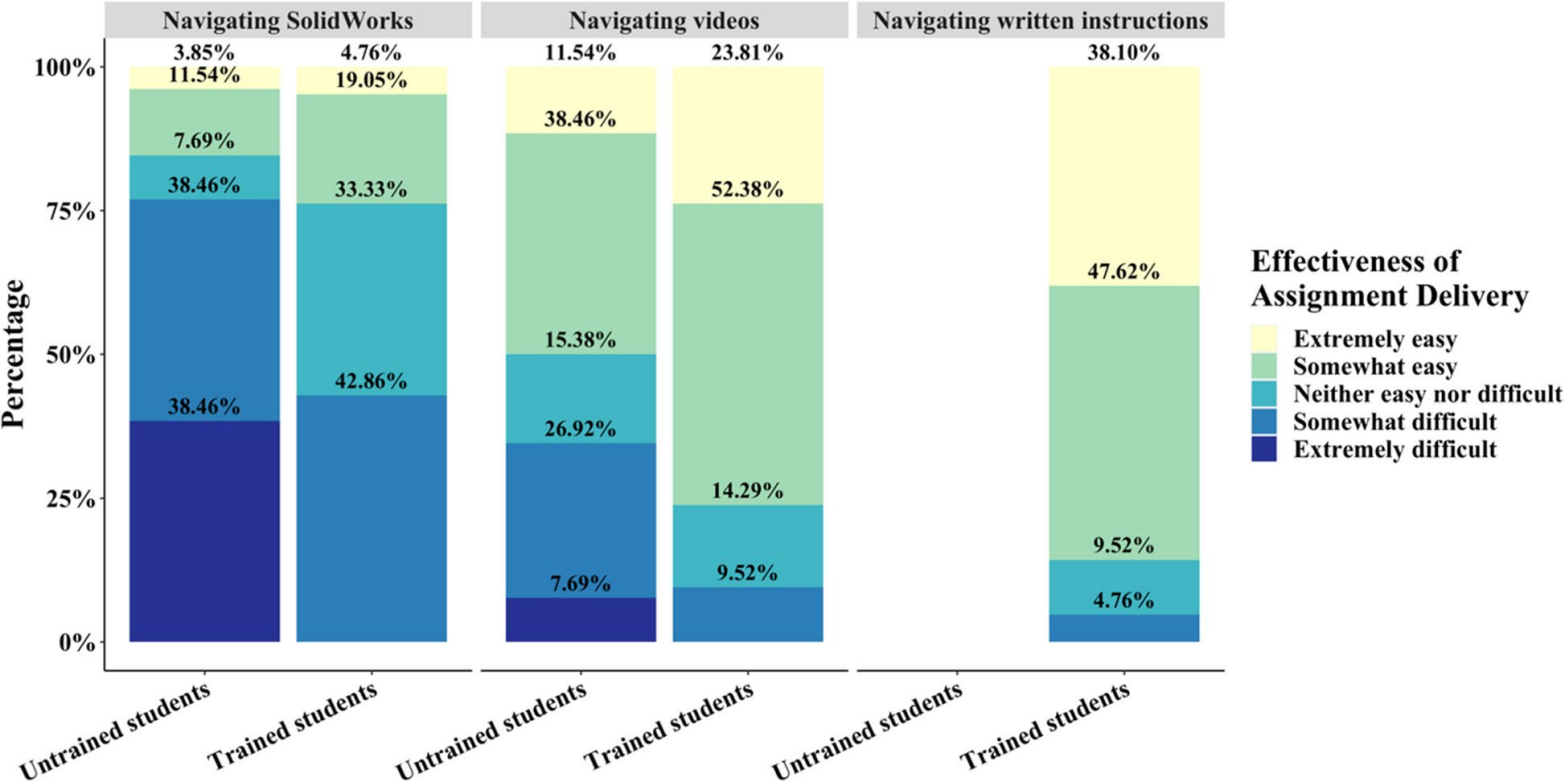
Likert plot of student responses in the post-assignment survey grading the ease with which the assignment could be navigated and interpreted for the untrained and trained student cohorts

Questions diagnosing the difficulty students had in completing and working through the assignment and the confidence of students in operating CAD-related software in the future were included to determine the effectiveness of the content delivery method. For the untrained group of students, while most responded that their ability to follow along with the demo videos was easy (50.0%), a large percentage of students also displayed difficulty in following along (34.61%), which may indicate that the videos need either a more in-depth tutorial or a slower delivery (Fig. 12). Furthermore, 76.92% of untrained students indicated that navigating SolidWorks was difficult (Fig. 12). The trained cohort of students, however, overwhelmingly considered navigating the demo videos extremely or somewhat easy with 76.91% of students providing a positive response while navigating SolidWorks was still majorly considered somewhat difficult by 42.86% of students (Fig. 12). Despite the majority of trained students still considering SolidWorks software navigation difficult, a statistically significant increase in ease of navigating SolidWorks was still detected between the trained and untrained cohort (*p* < 0.01). This significant increase was also present between the trained and untrained groups for ease of navigating demo videos (*p* < 0.05). Between scores for written instructions and video demos, there was no significant difference between ease of use if the trained group is only considered (*p* > 0.05). However, ignoring the potential confounding variable of written instructions, the trained and untrained score for navigating video demos is significantly lower than for navigating written instructions (*p* < 0.05).

**Fig. 13:**
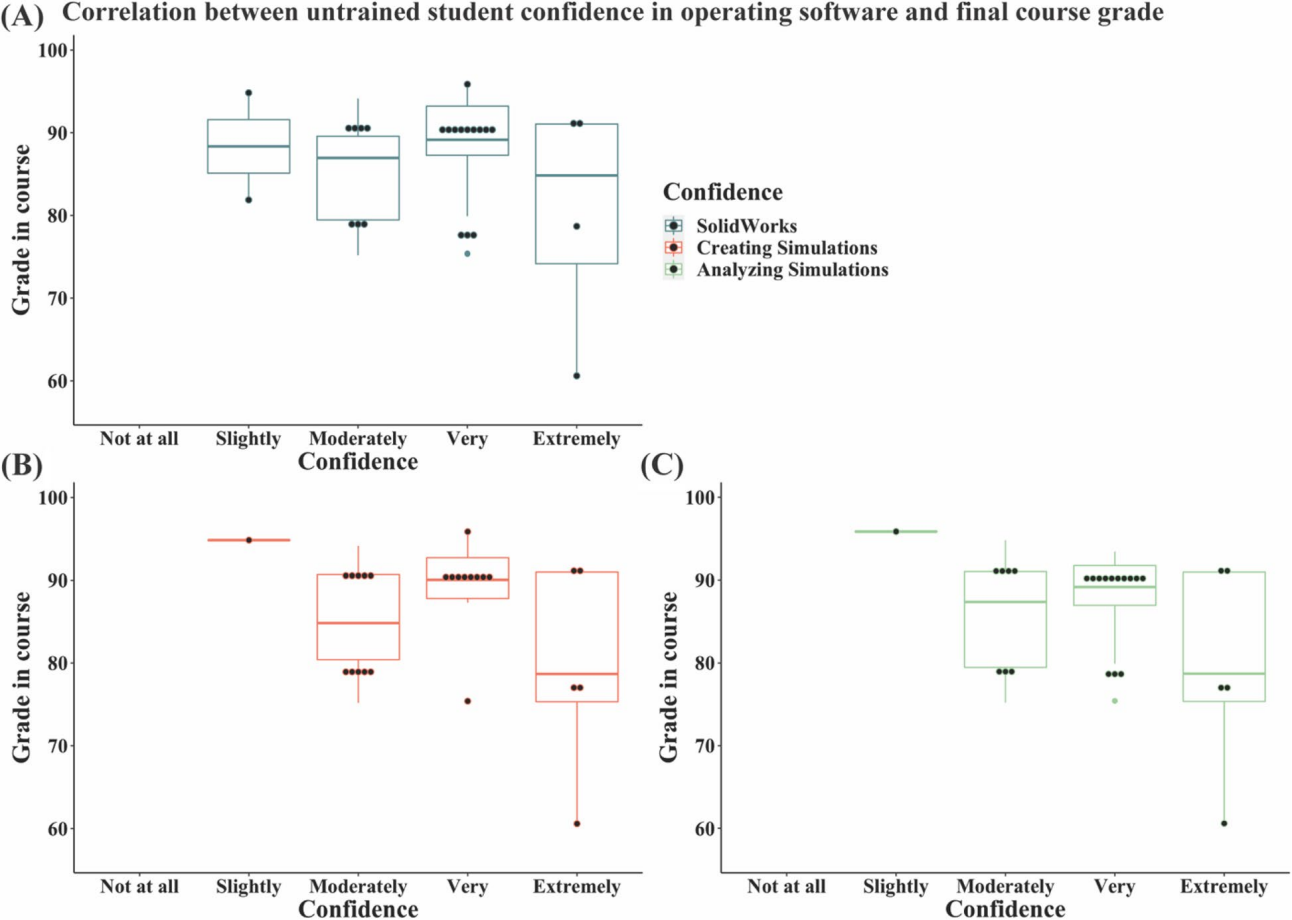
Distribution of student self-assessment of confidence assessed in the post-assignment survey in operating SolidWorks, creating simulations, and analyzing the resulting simulations from the post-assignment quiz for the untrained cohort. No significant correlation between student performance in the course and the confidence reported was detected

**Fig. 14:**
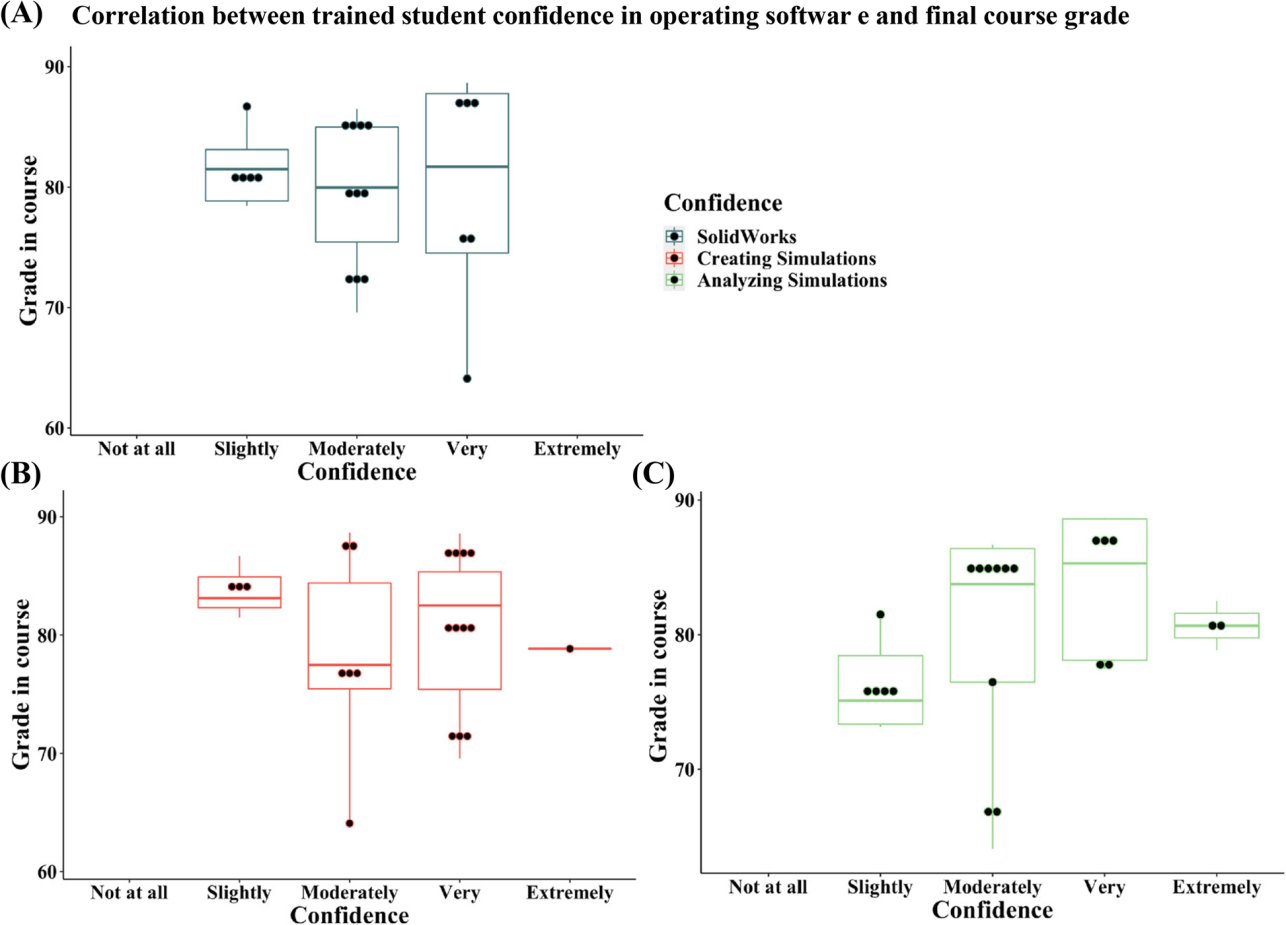
Distribution of student self-assessment of confidence assessed in the post-assignment survey in operating SolidWorks, creating simulations, and analyzing the resulting simulations from the post-assignment quiz for the trained cohort. No significant correlation between student performance in the course and the confidence reported was detected

To determine whether student performance in the course impacts their confidence in their ability to operate SolidWorks and SolidWorks Simulations, the grade distribution of each cohort was assessed for each self-assessed skill. Using linear regression analysis, correlation strengths between increasing confidence in SolidWorks, creating a simulation, and analyzing simulations and grades in the course were determined. For the untrained group, linear regressions detected no correlation between confidence in all groups and course grades (*p* > 0.05) (Fig. 13). Similarly, the trained group also had no significant correlation (*p* > 0.05), indicating that standing in the course did not influence confidence responses for 3D design skills (Fig. 14).

**Fig. 15:**
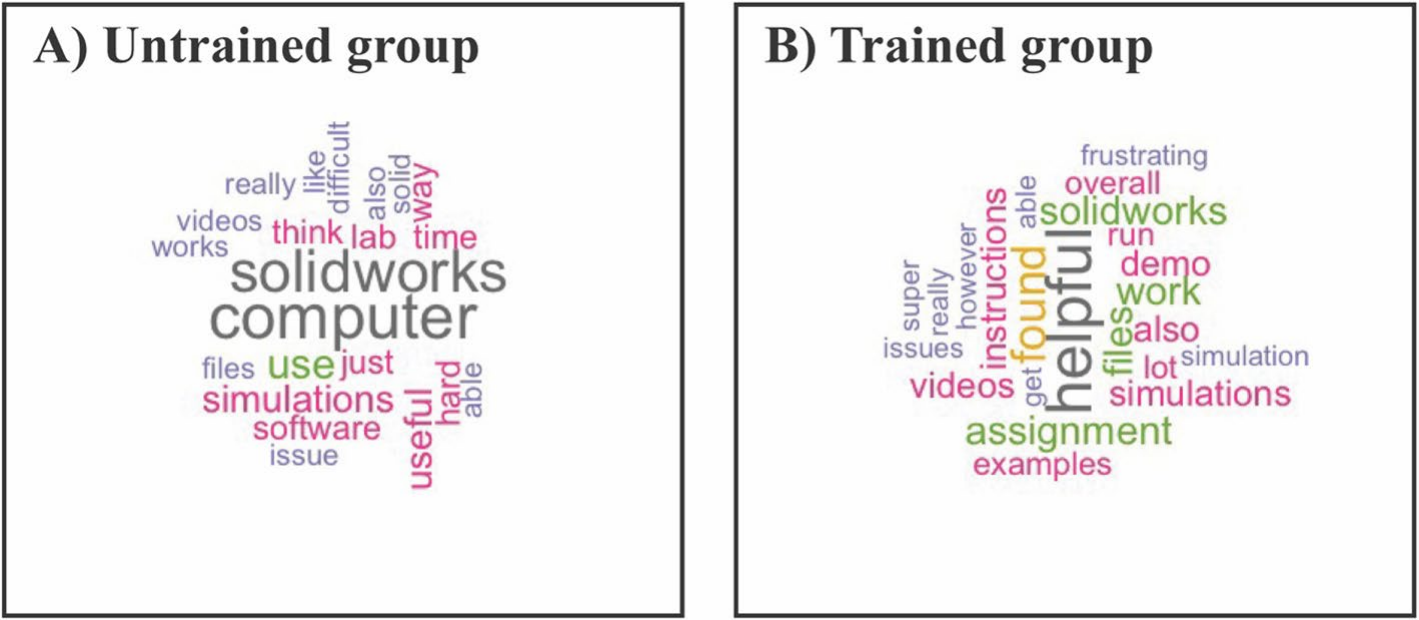
Word cloud of student open responses soliciting holistic feedback on the assignment in the post-assignment survey with a minimum frequency of 4 words detected to be present on the graphic

Based on the student feedback solicited after the completion of the assignment, the errors encountered were primarily due to long runtime, the inability to access SolidWorks through Citrix, and other technical difficulties in the untrained cohort (Fig. 15). The trained students also reported technical errors related to computer issues in running SolidWorks despite the low percentage of students responding positively to the question explicitly asking if errors were encountered (Fig. 11). Untrained students indicated that more detailed instructions within the assignment that addressed and developed countermeasures for these errors are needed and tended to focus on specific problems encountered in the assignment, which includes issues encountered with particular simulation studies due to either computational limitations or a lack of understanding relating to simulation restriction (Fig. 15). The second cohort of students also requested more detailed instructions focusing on the ineffectiveness of the demo videos alone (Fig. 15). Three students reported that the written instructions were a more effective delivery of assignment instructions and four students recommended that we reduce the number of simulations and increase the detail for each simulation’s set of instructions. However, the second cohort also overwhelmingly emphasized that this assignment effectively illustrated how helpful SolidWorks is (Fig. 15).

## Discussion

Student responses regarding previous experiences in 3D design or CAD-related software reflect the likeliness of students to independently study 3D design software. The student cohort without introductory training contained ~ 89% of students with no experience in any type of 3D design software (Fig. 6). Because an introductory module was provided to the second group, 100% reported some experience with 76.9% of students responding that they had experience with SolidWorks in particular (Fig. 6). Students are not likely to have used SolidWorks in their undergraduate career prior to their second semester sophomore (2nd) year, and, because downstream coursework does not include SolidWorks training (Fig. 1), students are not likely to actively use 3D design software within the BMEG curriculum. The incorporation of 3D design in undergraduate BMEG curricula is critical due to the broad applicability of 3D design and simulations in BMEG industries but is often excluded. Modern BMEG industries require students to understand and solve complex multi-faceted problems and training in modern manufacturing techniques, increases the student’s capacity to operate in such a field [15]. Teaching 3D design at an undergraduate level, further, has been previously demonstrated to improve student capacities to comprehend design approaches and develop professional skills [10, 16–18]. Consequently, adapting existing curricula to meet the need of providing students with the tools to succeed in a biomedical engineering industry is necessary.

The impact of initial training on the effectiveness and effect on student opinion of the assignment is exhibited in the results of the pre-and post-surveys. Previous training in basic SolidWorks part creation had no impact on students' self-assessed interest in SolidWorks pre- and post-assignment compared to the untrained group post-assignment (Fig. 7). These data indicate that the assignment did not differentially capture students’ interest in 3D design software and its usage between the two groups. Trained students, however, were more likely to express an ability to envision potential usage of SolidWorks simulations and 3D design in their future careers compared to the untrained group (Fig. 8). An increase in positive responses for both questions, however, indicates that the assignment did have a tangible impact on improving student opinion and perspectives on 3D design and simulations. The introductory course, in this instance, may be responsible for garnering an initial perspective on the applicability of SolidWorks through part design, which is amplified by providing application-based opportunities to practice SolidWorks skills. In the untrained group, however, extremely positive responses only emerged after the assignment was distributed (Figs. 7 and 8). A lack of prior knowledge of the capability of CAD-based software resulted in a more severe shift in opinion compared to the trained group.

Despite the positive student responses in likeliness to use and interest in SolidWorks post-assignment, the reported confidence in operating SolidWorks and SolidWorks Simulations was observed to be low in both the trained and untrained groups (Fig. 9). In the untrained cohort, approximately 60% of students expressed no or slight confidence in operating SolidWorks and analyzing SolidWorks Simulations while only around 30% in the trained group responded similarly, indicating that the trained group was more confident in their ability to operate SolidWorks and analyze simulations (Fig. 9). However, no students in either cohort reported extreme confidence in SolidWorks and SolidWorks Simulations, which is a reasonable assessment after only one or two modules of SolidWorks training. It is notable that while the trained cohort responded with a higher confidence in SolidWorks and analyzing simulations compared to the untrained group, this group also reported a majority low confidence in creating simulations. A significant lack in student confidence for the untrained group in SolidWorks itself is likely a product of a lack of experience in SolidWorks as a general 3D design tool (Fig. 9). Without familiarity with the software apart from the Simulations add-in, independently navigating and using SolidWorks can be difficult for students. Through the extra credit assignment, untrained students discovered that SolidWorks Simulations is a valuable tool that they are likely to use in their future careers, but a lack of basic training is a barrier in developing a reliable skill in this area. The trained cohort, while slightly more confident in their ability to run SolidWorks and analyzing the resulting simulations, did not gain confidence compared to the untrained group in creating simulations. These data indicate that the current content delivery method is not effective in providing students with a reliable skill in SolidWorks Simulations. However, the simulation toolkit was effective in illustrating the utility of SolidWorks Simulations independent of previous training as both the trained and untrained groups responded similarly that SolidWorks is indeed useful (Fig. 10).

The demo videos provided through the assignment were distributed in a Box file that students could additionally use to download the SolidWorks part/assembly and Simulations files. Based on the difficulty students had in navigating the assignment format (Fig. 12), a more user-friendly method of delivering the video content would improve students' ability to navigate the video files perhaps through a Blackboard page clearly providing links to each video. Blackboard is a web-based interface that all students use to access content for assignments and other information such as the syllabus for any given course. As a result, the graphical user interface is more user-friendly for navigating content and will be more familiar to the students. In the trained group, students preferred written delivery instructions compared to videos, which indicates that written supplements are useful for students completing this type of assignment. This is in contrast to recent educational research indicating that short-form videos are more effective for students [19–21]. However, as this is an instructional step-by-step, it is possible that allowing students to read through the instructions at their own pace was more beneficial. Concerns as to student retention and motivation to consume video-based instruction has also been previously raised and is a contentious subject in undergraduate education [22]. It is critical to note that provision of written instructions does serve as a confounding factor in the study, thus, it is possible that all differences in the post-survey could be a result of written instruction. Further, students from both cohorts were overwhelmed by the number of files that need to be downloaded prior to running the simulation. The high number of files is required because the pre-run simulation files must be downloaded with the parts or assemblies being analyzed for students to receive the solution. It may be more beneficial to provide the output requirements alone rather than the whole simulation result to improve the usability of the toolkit. Because the trained cohort in this study has already installed and run SolidWorks files through the introductory module, we anticipated that this issue would be preemptively solved through preliminary instruction. Students in the trained group did report fewer technical errors compared to the untrained cohort (Fig. 11) with more students including positive comments.

Linear regressions between final grades in the course and the student confidence reported for each 3D design and simulation skill demonstrate that confidence is not associated with in-class performance. Therefore, the assignment effectiveness was not dependent on whether the student was performing well in the class and could target students at all stages (Figs. 13 and 14). Student open responses revealed that the untrained group expressed more difficulty in managing and completing the assignment, while the trained group mostly complimented the software’s capabilities. Collectively, these data suggest that prior 3D design training is necessary for the assignment to impart simulation skills effectively.

## Conclusions

We designed a SolidWorks Simulations Toolkit that was developed for a sophomore (2nd-year) biomedical engineering course to enrich existing content with 3D design applications for students with and without explicit SolidWorks basic training. The Simulation Toolkit effectively demonstrated the benefits of SolidWorks Simulations with a sufficiently short tutorial that increased positive student opinion in both cohorts. The untrained group of students was more significantly impacted by the assignment with a more severe opinion shift post-assignment, but positive opinion was overall greater in the trained students. Despite an increased interest in and likeliness to use the software, student confidence in operating SolidWorks and SolidWorks Simulations remained low in both groups. Students with previous training felt more competent with SolidWorks but expressed a similar lack of confidence as the untrained group in SolidWorks Simulations. The assignment delivery method could be the cause of low confidence due to both groups reporting that the instructions and tutorials lacked sufficient detail that would facilitate their ability to extrapolate this skill elsewhere. However, notably, students in the trained cohort found navigating and completing the assignment easier compared to the untrained group, which was further reflected in an increase in positive comments. Without the initial training in part design and general SolidWorks tools, the assignment yields little tangible benefit for the untrained group aside from an increased interest in using SolidWorks in the future. Further, we were able to demonstrate that grade distribution had no correlation to confidence responses, which suggests that students at all skill levels were engaging with the assignment. These results are of interest for future investigations into students’ motivations for participating in the extra credit assignment.

This work confirms that implementing basic 3D design tutorials in 1^st^ and 2^nd^-year Biomedical Engineering courses improves student opinion of 3D design software and increasing student confidence in a marketable skill. Taken together, increased awareness of the utility of 3D design software and the ability to use the software can be improved through providing short basic tutorials followed by enriching existing courses with 3D design applications. However, the assignment delivery of the toolkit needs to be perfected such that the ease of assignment completion is increased compared to the current delivery method.

## Supplementary Information

**Additional file 1: Survey questions.**

## Data Availability

Authors will make the study data available upon request while adhering to the IRB protocol and the human subjects consent forms. The simulation files and assignment demos are also available in our GitHub repository: https://github.com/maryjia/BMEG2813-simulation-toolkit.

## Abbreviations

3D: Three-dimensional
2D: Two-dimensional
FEM: Finite Element Method
CAD: Computer-aided Design
BMEG: Biomedical Engineering

## References

[1] Kang H-W, Lee SJ, Ko IK, Kengla C, Yoo JJ, Atala A (2016). A 3D bioprinting system to produce human-scale tissue constructs with structural integrity. Nat Biotechnol.

[2] Miller JS (2012). Rapid casting of patterned vascular networks for perfusable engineered three-dimensional tissues. Nat Mater.

[3] Kolesky DB, Homan KA, Skylar-Scott MA, Lewis JA (2016). Three-dimensional bioprinting of thick vascularized tissues. Proc Natl Acad Sci.

[4] Guvendiren M, Molde J, Soares RMD, Kohn J (2016). Designing biomaterials for 3D Printing. ACS Biomater Sci Eng.

[5] Trenfield SJ, Awad A, Goyanes A, Gaisford S, Basit AW (2018). 3D printing pharmaceuticals: drug development to frontline care. Trends Pharmacol Sci.

[6] Trauner KB (2018). The emerging role of 3d printing in arthroplasty and orthopedics. J Arthroplasty.

[7] Harris TR, Bransford JD, Brophy SP (2002). Roles for learning sciences and learning technologies in biomedical engineering education: a review of recent advances. Annu Rev Biomed Eng.

[8] Banoriya D, Purohit R, Dwivedi RK (2015). Modern trends in rapid prototyping for biomedical applications. Mater Today Proc.

[9] Lantada AD, Morgado PL (2012). Rapid prototyping for biomedical engineering: current capabilities and challenges. Annu Rev Biomed Eng.

[10] Jia M, Crosby J, Rao R, Elsaadany M (2022). Integrating SolidWorks 3D Design and Simulation Modules into Introductory Biomedical Engineering Courses for the Development of Employability Skills.

[11] Robertson B, Radcliffe D (2006). The Role of Software Tools in Influencing Creative Problem Solving in Engineering Design and Education. Volume 4c: 3rd Symposium on International Design and Design Education.

[12] Howard TJ, Culley SJ, Dekoninck E (2008). Describing the creative design process by the integration of engineering design and cognitive psychology literature. Des Stud.

[13] Wang D, Lee J, Holland K, Bibby T, Beaudoin S, Cale T (1997). Von mises stress in chemical-mechanical polishing processes. J Electrochem Soc.

[14] Sullivan GM, Artino AR (2013). Analyzing and interpreting data from likert-type scales. J Grad Med Educ.

[15] Raman R, Mitchell M, Perez-Pinera P, Bashir R, DeStefano L (2016). Design and integration of a problem-based biofabrication course into an undergraduate biomedical engineering curriculum. J Biol Eng.

[16] Shaikh FUA (2012). Role of commercial software in teaching finite element analysis at undergraduate level: a case study. Eng Educ.

[17] Taleyarkhan M, Dasgupta C, Garcia JM, Magana AJ (2018). Investigating the impact of using a CAD simulation tool on students’ learning of design thinking. J Sci Educ Technol.

[18] Ebenstein D, Tranquillo J, Cavanagh D (2007). Developing Student Design And Professional Skills In An Undergraduate Biomedical Engineering Curriculum. 2007 Annual Conference & Exposition Proceedings.

[19] Zaneldin E, Ahmed W, El-Ariss B (2019). Video-based e-learning for an undergraduate engineering course. E-Learn Digital Media.

[20] Giannakos MN, Jaccheri L, Krogstie J (2016). How video usage styles affect student engagement? Implications for video-based learning environments.

[21] Long T, Logan J, Waugh M (2016). Students’ perceptions of the value of using videos as a pre-class learning experience in the flipped classroom. TechTrends.

[22] McNulty JA (2009). An analysis of lecture video utilization in undergraduate medical education: associations with performance in the courses. BMC Med Educ.

