# Supplementary material for: Early introduction of 3D modeling modules promotes the development of simulation skills in downstream biomedical engineering curricula
Source: J Biol Eng. 2023 Mar 30;17:26. doi: 10.1186/s13036-023-00339-7 (PMC10064548; doi:10.1186/s13036-023-00339-7)
Supplement: Supplementary file 1 — Additional file 1: Survey questions. [file 13036_2023_339_MOESM1_ESM.docx]

**Supplementary data – survey questions**

Q1 First and Last Name

________________________________________________________________

Q2 Email address (ex. registra@uark.edu)

________________________________________________________________

Q3 Do you have previous experience in design (CAD or otherwise)?

- Yes (1)
- No (2)

Q4 Do you have previous experience in CAD programs?

- Yes (1)
- No (2)

Q5 Do you have previous experience in SOLIDWORKS?

- Yes (1)
- No (2)

Q6 Do you have previous experience using SOLIDWORKS Simulation?

- Yes (1)
- No (2)

Q7 How many of the videos did you watch?

- 0 (0)
- 1 (1)
- 2 (2)
- 3 (3)
- 4 (4)

Q8 How difficult was it to navigate through SOLIDWORKS?

- Extremely easy (1)
- Somewhat easy (2)
- Neither easy nor difficult (3)
- Somewhat difficult (4)
- Extremely difficult (5)

Q9 How difficult was it to follow along with the instructional videos?

- Extremely easy (1)
- Somewhat easy (2)
- Neither easy nor difficult (3)
- Somewhat difficult (4)
- Extremely difficult (5)

Q19 How difficult was it to follow along with the instructional tutorials?

- Extremely easy (1)
- Somewhat easy (2)
- Neither easy nor difficult (3)
- Somewhat difficult (4)
- Extremely difficult (5)

Q10 Were you able to replicate the simulation analyses?

- Yes (1)
- No (2)

Q11 Were you able to analyze and compare your results?

- Yes (1)
- No (2)

Q12 Did you encounter any kind of error?

- Yes (1)
- No (2)

Q13 Before or after viewing any content, how interested were you in CAD software (SOLIDWORKS)?

|  | Extremely (1) | Very (2) | Moderately (3) | Slightly (4) | Not at all (5) |
| --- | --- | --- | --- | --- | --- |
| BEFORE |  |  |  |  |  |
| AFTER |  |  |  |  |  |

Q14 How confident are you in the following?

|  | Extremely (1) | Very (2) | Moderately (3) | Slightly (4) | Not at all (5) |
| --- | --- | --- | --- | --- | --- |
| SOLIDWORKS (1) |  |  |  |  |  |
| Creating Simulations (2) |  |  |  |  |  |
| Analyzing Simulations (3) |  |  |  |  |  |

Q15 How useful is SOLIDWORKS Simulation?

- Extremely useful (1)
- Very useful (2)
- Moderately useful (3)
- Slightly useful (4)
- Not at all useful (5)

Q16 How likely were you to use SOLIDWORKS before and after?

|  | Extremely likely (1) | Somewhat likely (2) | Neither likely nor unlikely (3) | Somewhat unlikely (4) | Extremely unlikely (5) |
| --- | --- | --- | --- | --- | --- |
| BEFORE (1) |  |  |  |  |  |
| AFTER (2) |  |  |  |  |  |

Q17 Please provide any comments or suggestions. Please elaborate and be detailed.

________________________________________________________________
